# Fluorescent Nanosystems for Drug Tracking and Theranostics: Recent Applications in the Ocular Field

**DOI:** 10.3390/pharmaceutics14050955

**Published:** 2022-04-28

**Authors:** Elide Zingale, Alessia Romeo, Salvatore Rizzo, Cinzia Cimino, Angela Bonaccorso, Claudia Carbone, Teresa Musumeci, Rosario Pignatello

**Affiliations:** 1 Department of Pharmaceutical and Health Sciences, University of Catania, 95124 Catania, Italy; elide.zingale@gmail.com (E.Z.); alessia.romeo@phd.unict.it (A.R.); salvo_rizzo@outlook.it (S.R.); cinzia.cimino@phd.unict.it (C.C.); angela.bonaccorso@unict.it (A.B.); ccarbone@unict.it (C.C.); teresa.musumeci@unict.it (T.M.); 2 NANO-*i*—Research Center for Ocular Nanotechnology, University of Catania, 95124 Catania, Italy

**Keywords:** nanotechnology, fluorescence, ocular delivery, probes, diagnostics, PKs

## Abstract

The greatest challenge associated with topical drug delivery for the treatment of diseases affecting the posterior segment of the eye is to overcome the poor bioavailability of the carried molecules. Nanomedicine offers the possibility to overcome obstacles related to physiological mechanisms and ocular barriers by exploiting different ocular routes. Functionalization of nanosystems by fluorescent probes could be a useful strategy to understand the pathway taken by nanocarriers into the ocular globe and to improve the desired targeting accuracy. The application of fluorescence to decorate nanocarrier surfaces or the encapsulation of fluorophore molecules makes the nanosystems a light probe useful in the landscape of diagnostics and theranostics. In this review, a state of the art on ocular routes of administration is reported, with a focus on pathways undertaken after topical application. Numerous studies are reported in the first section, confirming that the use of fluorescent within nanoparticles is already spread for tracking and biodistribution studies. The first section presents fluorescent molecules used for tracking nanosystems’ cellular internalization and permeation of ocular tissues; discussions on the classification of nanosystems according to their nature (lipid-based, polymer-based, metallic-based and protein-based) follows. The following sections are dedicated to diagnostic and theranostic uses, respectively, which represent an innovation in the ocular field obtained by combining dual goals in a single administration system. For its great potential, this application of fluorescent nanoparticles would experience a great development in the near future. Finally, a brief overview is dedicated to the use of fluorescent markers in clinical trials and the market in the ocular field.

## 1. Introduction

In recent years, vision-related problems have acquired a greater relevance due to the ageing of the world’s population, which leads to an increase in visual problems, such as cataracts, glaucoma, age-related macular degeneration and diabetic retinopathy, occurring more frequently among over-60s [1,2]. Many visual diseases are associated with neurodegenerative disorders [3,4]. Young people over the age of 18 also suffer from visual problems, which increase especially with the growing use of electronic devices [5]. The rising number of people with vision impairment leads to a greater interest in dedicated care and treatments. This situation increases the costs in the global economy destined for the care of these disorders [6]. In addition, ocular therapy is a serious challenge because of the difficulty in targeting a drug to the appropriate ocular tissues.

In this landscape, technological research is actively involved, with the aim of developing innovative systems for targeted drug delivery [7]. The eye is a very complex structure, both anatomically and physiologically, and the treatment of pathologies affecting this organ is therefore not simple [8,9,10]. This is related to the various aspects that limit the transportation of drugs to the target site: anatomical barriers, physiological processes, mechanisms and metabolic aspects [11,12]. Reaching the target becomes more complicated if therapy is addressed to the posterior segment of the eye [13,14,15,16]. For this purpose, the major administration route remains intravitreal injection, which is invasive and produces undesirable effects such as pain and discomfort, inducing patient noncompliance [17,18]. The preferred route of administration would undoubtedly be the topical one, but conventionally it is used to treat diseases of the anterior eye. In fact, it is estimated that only a very small percentage of the drug instilled in the eye surface reaches the anterior chamber (around 5%) and even less in the posterior segment [19,20,21].

Nanotechnology represents a field of recent interest to overcome these issues. One potential strategy for improving drug delivery to the different eye tissues uses nanocarriers with specific size and surface properties, designed to ensure successful achievement of the drug to the target tissue, as well as the potential for a controlled release of the loaded drug, reducing the frequency of treatment and improving the retention time on the corneal surface [22,23,24]. Currently, the most widely studied nanosystems are used in the treatment of anterior eye diseases such as cataracts [25], glaucoma [26], dry eye syndrome [27], keratitis [28], conjunctivitis [29] and uveitis [30], but also posterior eye diseases such as retinitis [31], macular degeneration [32], endophthalmitis [33] and ocular tumors [34]. Suitable drug nanocarriers possess a mean size in the nanometric range (around 200 nm) and are classified according to their structural composition and the materials used, which must be biodegradable and biocompatible [35,36]. Many reviews focus on the development of nanosystems designed for ocular delivery, but none on the ophthalmic use of fluorescent nanocarriers. It is not certain that after their administration, the drug effectively reaches the target site; therefore, during its design, tracking studies are necessary to demonstrate its distribution and positioning.

One possible strategy is to follow the nanosystem movements using a fluorescent probe. Fluorescence is a simple and non-invasive way to track the drug through the eye tissues, and it is also widely used in diagnostics to visualize diseased tissues, lesions and pathological markers. The development of personalized medicine and the need for early intervention in the diagnosis and treatment of specific diseases have promoted the birth and development of a new discipline: theranostics [37]. It can be defined as the combination of diagnostics with a specific therapeutic treatment. In vitro diagnostics and prognostics, in vivo molecular imaging, molecular therapeutics, image-guided therapy, biosensors, nanobiosensors and bioelectronics, system biology and translational medicine and point-of-care are some recent application examples.

This review deals with the use of fluorescent probes in the last 5 years applied to nanomedicine in the ophthalmic field. The aim is to illustrate state-of-the-art fluorescent nanosystems divided according to their application: fluorescent nanosystems for biodistribution studies to clarify the best performing nanoparticle design and delivery strategies able to address specific ocular diseases, for diagnostics and finally, for the emerging field of theranostics. PubMed database was used to perform an advanced search. The time frame included the range from January 2017 to February 2022. The keywords used were “fluorescence”, “nanoparticles”, “ocular” and “delivery”, “theranostics”, “diagnostics”. Articles were limited to “Free full text” and “Full text” articles in the English language published in journals with an impactor factor not less than 4. The same process was repeated on ScienceDirect database. Reference lists of articles were also reviewed for additional citations.

### General Aspect of the Human Eye

**Figure 1:**
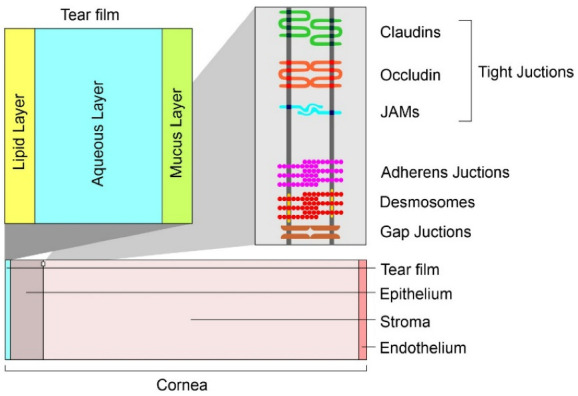
Cross-section of corneal tissues: barriers to drug penetration after topical instillation.

The eyeball consists of three chambers: anterior, posterior (containing the aqueous humor) and the vitreous chamber (containing the vitreous body). The wall is composed of three tunics [8,38]. The first, called external, is composed anteriorly of the cornea and for the remaining part of the sclera. The middle tunic (uvea) is richly vascularized and pigmented and includes the iris, the ciliary body and the choroid. Finally, the internal or nervous tunic is represented by the retina [39]. The sclera is anteriorly lined by the conjunctiva. Its function is to maintain the shape of the bulb and to provide attachment to the tendons of the striated muscles of the eye [40]. The cornea is a transparent lamina without vessels (necessary conditions for the passage of light). A cross-section of the corneal tissues is shown in Figure 1. Under the cornea, there is the iris, a sphincter of pigmented smooth muscle that regulates pupillary caliber. Trophism in this district is provided by the aqueous humor [41]. The ciliary body is an ocular anatomical structure responsible for both the production of aqueous humor and the control of accommodation. The ciliary body is located immediately posterior to the iris and anterior to the choroid. Posterior to the iris and in front of the vitreous body is where the crystalline is situated, which transmits and focuses light onto the retina. It consists of a single layer of epithelial cells that, during fetal development, migrate laterally toward the equator of the lens where it inverts, elongates, synthesizes large amounts of specific proteins and finally, degrades organelles so as to increase transparency [20]. From a physiological perspective, there are two reflexes involved in vision: lens accommodation (regulates convexity) and pupillary reflex (regulates pupil caliber). The accommodation allows the focal point to fall always at the level of the retina, allowing both short- and long-distance vision. Furthermore, the pupillary reflex regulates the intensity of incoming light. Finally, the transduction of light impulses at the retinal level into visual images is mediated by photoreceptors which generate nerve stimuli that reach the contralateral posterior cortex through the optic nerve [42,43,44]. The delivery of a drug into the eye tissues is related to two different routes of administration, which are divided into invasive and non-invasive routes. A list of these routes is shown in Table 1.

The corneal epithelium and endothelium (lipophilic in nature) consist of cells connected by tight junctions that limit the passage of large molecules (Figure 1). The hydrophilic stroma consists of tightly packed collagen. The epithelium, however, provides the greatest resistance to diffusion. The paracellular pathway through the intercellular pores is allowed for small ionic and hydrophilic molecules of size <350 Da, whereas the transcellular pathway allows the passage of larger lipophilic molecules. The variations in lipophilicity of the corneal layers allowed the realization of a parabolic relationship between corneal permeability and diffusion coefficient. pH is another important factor in corneal permeability [38]. Many studies that have examined permeability across conjunctiva, tenon and sclera have shown that the conjunctiva is more permeable to hydrophilic molecules than the cornea. The greater surface area (in humans, about 17 times bigger than the cornea) and the presence of larger pore sizes promote increased permeability compared to the cornea. However, mucus and the presence of lymphatics and vasculature increases systemic leakage [24,38]. In ocular topical administration, reaching the posterior portion is size-dependent [65]. Nanocarriers with a diameter of 20–200 nm are suitable for retinal-targeted delivery. Small nanoparticles (20 nm) are able to cross the sclera and are rapidly eliminated due to periocular circulation. The larger ones (200 nm) do not cross the sclera or the sclera-choroid-retinal pigment epithelium (RPE) and remain in the periocular site releasing their contents even for long periods. Even in the case of intravitreal administration, the kinetics are size-dependent. Nanocarriers with a diameter of 2 μm remain in the vitreous cavity or migrate into the trabeculae. Those with a diameter of less than 200 nm reach the retina [66]. In order to discuss the application of nanosystems in the ocular field, an emergent role is represented by fluorescent nanosystems. The tailor ability of design, architecture and photophysical properties has attracted the attention of many research groups, resulting in numerous reports related to novel nanosensors to analyze a great variety of biological analytes.

## 2. Fluorescent Probes in Ocular Applications

Before focusing on the published experimental studies, in this section, a brief discussion on fluorescence and on the molecules applied in the ocular field is given.

Absorption of a photon from a fluorescent chemical species causes a transition to an excited state of the same multiplicity (spin) as the fundamental state (S0). In solution, Sn states (with n > 1) rapidly relax to S1 through nonradiative processes. Ultimately, relaxation from S1 to S0 causes the emission of a photon with an energy lower than the absorbed photon. The fluorescence quantum yield (φ), one of the most important parameters, provides the efficiency of the fluorescence process; it is defined as the ratio between the number of photons emitted to those absorbed.

$$\varphi =\frac{Number\;of\;photons\;emitted}{Number\;of\;photons\;absorbed}\;$$

**Figure 2:**
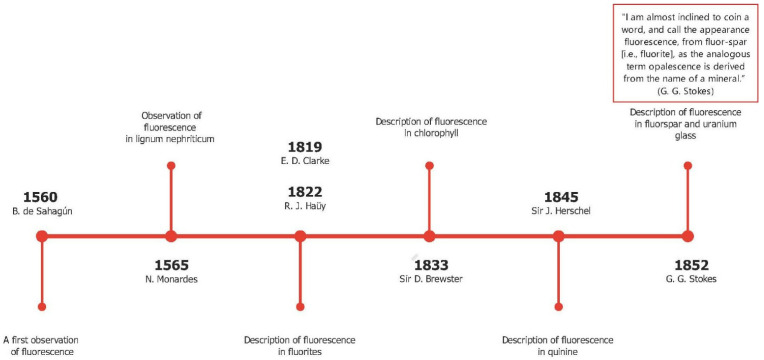
Timeline of the fluorescence discovery.

In Figure 2, we reproduce a brief history of the discovery of the fluorescence phenomenon. This discovery enabled the development of fluorescent probes that achieve single-molecule sensitivity. The figure shows that the first observation of a fluorescence phenomenon was described in 1560 by Bernardino de Sahagun; the same experiment was repeated by Nicolas Monardes in 1565. The fluorescence of the infusion known as lignum nephriticum was observed. This phenomenon was caused by the fluorescence of the oxidation product of one of the flavonoids present in those woods: matlaline. In the middle of the nineteenth century, George Gabriel Stokes coined the term fluorescence, derived from fluorite. The knowledge of atomic structure needed to understand and describe the nature of the phenomenon was not acquired until the beginning of the 20th century. By providing detailed information, this technique has enormous advantages over classical microscopy techniques [67]. In fact, literature is plentiful of studies dealing with the design of new fluorescent probes such as (bio)sensors to detect (even with the naked eye) enzymes, metals, biomaterials and others. Since 1945, the ability of analytes to promote the opening of rhodamine spirolactams has been exploited to design probes that detect metal ions and biological targets [68,69]. The pH sensitivity of fluorescein can be used to detect changes in a specific environment. By controlling the balance of ring-opening and ring-closing, following the interaction with specific targets, it can be used to detect metal ions from industrial and commercial specimens [70]. Curcumin is also widely used as a fluorescent probe for different applications, from producing drug carriers to the realization of specific sensors for ions and biomolecules [71,72].

The following section delineates the family of fluorescent probes reported in reviewed studies, while Table 2 gathers the probes that are used in the experimental papers cited in this review.

### 2.1. The Coumarins Family

Coumarins have a conjugated double ring system. In the industry, coumarins find application as cosmetic ingredients, perfumers, food additives and in synthetic pharmaceuticals. In nature, coumarins are found in a wide variety of plants: tonka bean (*Dipteryx odorata*), sweet wood (*Galium odoratum*), vanilla grass (*Anthoxanthum odoratum*) and sweet grass (*Hierochloe odorata*) [73]. Among the different synthetic derivatives, Coumarin-6 (C6) exhibits acid-base properties. In the study of Duong et al., a membrane with C6 demonstrated to exhibit colorimetric and ratiometric fluorescence properties with a dynamic pH range between 4.5 and 7.5 (the study uses blue nile in parallel) [74].

### 2.2. Fluorescein Family

Fluorescein is a xanthene dye with yellowish-green fluorescence. It was firstly synthesized in 1871 by von Bayer via Friedel’s acylation/cyclodegradation reaction using resorcinol and phthalic anhydride [75]. It has a rigid tricyclic-coplanar structure with two aryl groups fused to a pyran ring. It has two distinct structures, an open fluorescent ring in the carboxylic acid form and a closed non-fluorescent ring in the spirocyclic lactone form. The open-closed equilibrium in the structure of fluorescein makes it sensitive to the pH of the medium [76]. Among the amine derivatives of fluorescein, those with one or two NH_2_ groups in the phthalic residue are of particular interest. The corresponding (di)anions do not show intense fluorescence unless the amine groups are involved in new covalent bonds. In alcohols, the quantum yield, *φ*, is quite low. In dimethylsulfoxide (DMSO), acetone and other hydrogen bond donor solvents, *φ* values approach dianionic values [77]. Its sodium salt form finds wide use in angiography [78,79] and glioma studies [80]. Fluorescein 5(6)-isothiocyanate has been used for fluorescence labeling of bacteria, exosomes, proteins (immunofluorescence) and H Protein for gel chromatography. The 5-(iodoacetamido)-fluorescein is used for the synthesis of fluorescently labeled organelles, proteins, peptides and enzymes. Finally, the 5(6)-carboxyfluorescein, a fluorescent polyanionic probe, was used to measure changes in intracellular pH and to highlight processes such as dendrimer aggregation and absorption [81].

### 2.3. Rhodamine Family

These compounds were discovered in 1887. In the 4–10 pH range, their fluorescence spectra are unaffected by changes. The typical chemical structure of rhodamines involves three benzene rings, whose spirocyclic/open-ring conversion results in their off/on fluorescence [82]. In nonpolar solvents, they exist as spironolactone forms with very low *φ* due to disruption of p-conjugation of the xanthene core. In polar solutions, the lactone form undergoes charge separation to form a zwitterion [68]. In open-loop forms, rhodamine dyes exist as ammonium cations that can be driven into mitochondria via MMP (Matrix MetalloProteinase). A famous example is rhodamine 123, which forms the basis of the Mito-Tracker dye [83]. Lastly, the rhodamine 6G is a rhodamine analog useful in Pgp (P-glycoprotein) efflux assays, and it has been used to characterize the kinetics of MRP1 (multidrug resistance protein 1)- mediated efflux. An in vivo study of rhodamine B-labeled polymeric nanoparticles was conducted by Bonaccorso et al. to evaluate the distribution in brain areas after intranasal administration of the formulation [84].

### 2.4. Cyanine Family

Cyanine dyes are among the most widely used families of fluorophores. Cyanine 5 (Cy5) has five carbon atoms in the bridge. It becomes reversibly photocommutable between a bright and dark state in the presence of a primary thiol [85]. Cy5 excited by visible light undergoes thiolation with a thiol anion and transforms into a non-fluorescent thiolated Cy5. The thiolated Cy5 returns to the light-emitting dethiolated form simply by UV irradiation [86]. The photophysical properties of organic dyes with rotatable bonds are strongly governed by their internal rotation in the excited state since rotation can greatly affect molecular conformation and bond conjugation [87]. In the biological field, it finds use in comparative genomic hybridization, transcriptomics in proteomics, and RNA localization [88]. Moreover, DiI is a cyanine-derived dialkyl carbon sensitive to the polarity of the environment. It is weakly fluorescent in water but highly fluorescent in nonpolar solvents. It is commonly used as a lipophilic marker for fluorescence microscopy in the biological field. DiI molecules penetrate in cell membranes with the 2 long alkyl chains (12 carbons) immersed in the bilayer and the rings parallel to the bilayer surface. The dye emits characteristic bright red fluorescence when its alkyl chains are incorporated into membranes making it particularly useful for tracking in the biological membrane [89]. In the study by Musumeci et al. the 1-1′-dioctadecyl-3,3,3′,3′-tetramethylindotricarbocyanine iodide dye was used to label polymeric nanoparticles and study their cerebral delivery after intranasal administration [90].

### 2.5. Nile Red

Nile red is a hydrophobic dye of recent interest in the identification of microplastics [91]. It is widely used in biophysical studies focusing on proteins, lipids and live-cell analysis. Depending on the environment, Nile Red shows different absorption and fluorescence spectra. In particular, in organic solvents or nonpolar environments, it shows strong fluorescence that changes depending on the environment, presenting shifts toward blue emission in nonpolar environments [92].

### 2.6. Curcumin

Curcumin is the main natural polyphenol found in the rhizome of *Curcuma longa* (turmeric) and in others *Curcuma* spp. Its countless benefits in the treatment of inflammatory states, metabolic syndrome, pain and inflammatory-degenerative conditions of the eyes are related to its antioxidant and anti-inflammatory effects [93]. Theoretical studies have predicted that its wide absorption band (410 and 430 nm) is due to the π-π* transition, while the maximum absorption between 389 and 419 nm is related to the keto and enol form, respectively [67].

### 2.7. Toluidine Blue O

Toluidine blue (TB) is a thiazine-based metachromatic dye. It has a high affinity for acidic tissue components. This characteristic allows colorimetric identification of DNA- and RNA-rich tissues [94]. In the ocular field, Navahi et al. performed a study on the use of TB in the diagnosis of ocular surface squamous neoplasm (OSSN) [95]. In the Su et al. study, in vivo antibacterial efficacy of TB-mediated photodynamic therapy on bacterial keratitis by *Staphylococcus aureus* in a rabbit was demonstrated. This provides a new option for the clinical treatment of bacterial keratitis [96].

## 3. Fluorescent Nanosystems in Ocular Application

**Figure 3:**
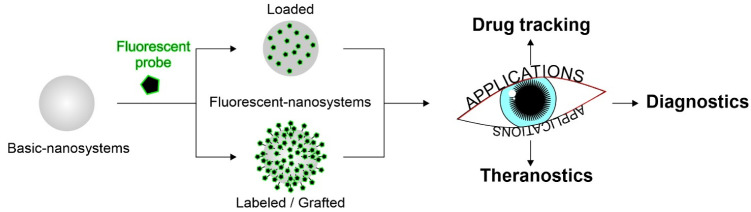
Schematic structure of fluorescent nanosystems for ocular applications.

The following section is focused on recently investigated fluorescent nanomaterials and nanosystems for ocular applications. The reviewed works have been divided according to the use of such fluorescent nanosystems. Most studies concern the use of probes to assess nanosystems distribution within the ocular tissues. Among the most investigated fluorescent nanosystems, there are lipid-based nanocarriers—such as nanostructured lipid carriers (NLCs) and solid lipid nanoparticles (SLNs), polymeric nanoparticles and nanocapsules, hybrid nanoparticles, cubosomes, emulsomes, nanoemulsions, niosomes, liposomes, films, nanomicelles and hydrogels. Fluorescence is introduced through the methods commonly used to prepare nanosystems [97,98]. The fluorescent nanosystems are essentially divided into (i) probe-loaded, in which the dye or probe is encapsulated into the system mostly during the formulation processes, and (ii) labeled/grafted, in which the probe is covalently bound to the surface of the nanosystem (often linked to some matrix component, such as polymers or lipids), always forming an adduct (Figure 3).

### 3.1. Biodistribution

**Figure 4:**
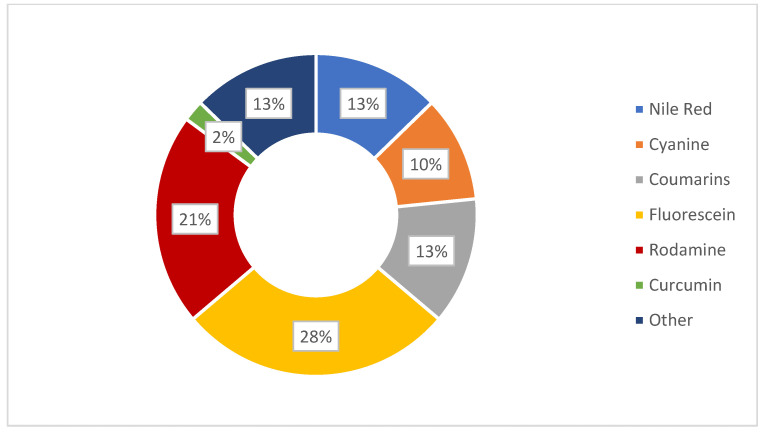
Graphical analysis of the fluorescent probes discussed in this review.

As above cited, the tissues that compose the eye are many and with different properties. The difficulty for a nanosystem to reach the target tissue is high; thus, the profile of drug delivery is not always predictable. When the target is located in deeper ocular tissues, it is even more difficult to predict the ideal pathways followed by the carriers in vivo and through the ocular barriers. Tracking the drug after topical administration is important for several factors. Firstly, it allows for assessment of the effective achievement of the target site in order to accomplish the desired therapeutic action. Another factor to consider is the non-productive distribution of the drug in non-desired tissues, which could lead to the possible occurrence of side effects in addition to reducing the effective drug concentration. Furthermore, studying the pathways followed by the nanosystems is necessary to avoid issues related to barriers, tight junctions and physiological phenomena (tear flow and blinking), which could impair the routes. Size, surface charge and morphology of the nanocarriers have a great influence on their biodistribution, clearance and cellular uptake [99,100,101,102]. Before performing biodistribution studies, it is important to characterize the system and to proceed with in vitro and in vivo assays. For instance, mean size measurement, zeta potential, mucoadhesion studies and morphological analyses are, of course, also required to make the system as conformable as possible to a correct drug release. Tracking of nanosystems can be carried out in two ways, invasive and non-invasive; bioimaging using fluorescent molecules is a non-invasive method [103,104]. Among the most important characteristics that the nanosystem should have are small size, necessary to enter cells for allowing bioimaging, high sensitivity for effective detection, fast response, compatibility, absence of toxicity, good dispersibility in the biological environment and highly selective detection in the tissues. In Figure 4, a summary is gathered of the fluorescent probes used in the studied nanosystems discussed in Section 3.1, Section 3.2 and Section 3.3

#### 3.1.1. Fluorescent Lipid-Based Nanosystems

Lipid systems are of great interest for drug delivery in ocular tissues; their biocompatible and biodegradable composition makes them technologically safe, while their lipidic nature and structural characteristics allow them to pass through the corneal layers and achieve an efficient drug dosage even in the deepest tissues of the eye. The distribution of these systems occurs mainly in lipophilic layers, with minimal involvement of the stroma, since it has hydrophilic nature, and the lipid systems are difficult to distribute there. This was confirmed in the work of Namprem et al., in which confocal scanning microfluorometer (CSMF) analysis confirmed poor penetration of NLC labeled with Nile Red in hydrophilic compartments such as the stroma compared to corneal layers [105]. Due to eye barriers and obstacles to ocular administration, understanding the path taken by the designed nanosystem is necessary, especially if it is targeted to the back of the eye. The main route through which lipid systems reach the deeper tissues is the transcorneal one. There is growing evidence that successful drug delivery by functionalized nanocarriers depends largely on their efficient intra/paracellular transport, a process that is not fully understood yet. Therefore, the development of new imaging and diagnostic techniques is very important, particularly in a complex biological system such as the eye. Due to its lipophilic nature, one of the most used dyes for the preparation of fluorescent-lipid nanosystems is Nile Red (NR) Cubosomes labeled with Nile Red were prepared in the work of El Gendy et al. to assess the role of nine different lipids as penetration enhancers. The type of lipid used in the preparation plays an important role in tissue distribution. Among the prepared lipid systems, fluorescence analysis showed that the combination of oleic acid, Captex^®^ 8000 and Capmul^®^ MCM improved the penetration of the systems into the mucosa by increasing diffusivity due to both surfactant properties and the ability to disrupt the organization of the lipid bilayer [106]. Once again, Nile Red was used in the work of Kapadia et al. in order to visualize drug-loaded emulsomes. For the physico-chemical characterization and subsequent analyses, the nanosystems were loaded with triamcinolone acetonide, while for the studies of precorneal retention and ocular distribution, the fluorescent dye was loaded instead of the drug. The study revealed that after topical administration, the pathways taken to reach the back of the eye were basically three: corneal (via the iris and aqueous humor), conjunctival and systemic. The drug may diffuse through the sclera by lateral diffusion, followed by penetration of Bruch’s membrane and retinal pigment epithelium (RPE). To a lesser extent, the drug may be absorbed into the systemic circulation either through the conjunctival vessels and the nasolacrimal duct, and gain systemic access to the retinal vessel [107]. Another lipophilic DiI dye (1,1-dioctadecyl-3,3,3 tetramethyl indocarbocyanine perchlorate) was used to label lipid nanocapsules (LNCs) fluorescently. An important finding was made in the study by Eldesouky et al., where, despite the lipophilic nature of the dye, better penetration was achieved by encapsulation in lipid systems compared to simple dispersion. Fluorescence analysis showed that, without the use of lipid nanocarriers, the dye is unable to cross the hydrophobic corneal layer [108]. Mucoadhesion plays a key role in the enhancement of bioavailability. Efforts are made to design systems that have the ability to improve retention on the ocular surface. In this respect, the use of chitosan to improve the delivery of drugs into the eye tissues for its properties as a mucoadhesive agent, controlled drug release and permeation enhancer is interesting [24]. It is used in conjugation with a drug, such as in the study of Dubashynskaya et al., to improve the intravitreal delivery of dexamethasone [109]. In the major cases, it was used as a coating of nanocarriers to promote intraocular penetration, as reported by which designed modified NLCs with three different types of chitosan: chitosan acetyl-L-cysteine (CS-NAC), chitosan oligosaccharides (COS) and carboxymethyl chitosan (CMCS). The distribution profile was evaluated by loading the hydrophobic dye C6 into the NLCs. It was revealed through CLSM analysis that only NLCs modified with COS and CS-NAC were able to pass the cornea through the opening of tight junctions between epithelial cells [110]. Rhodamine-labeled NLCs were used to assess the corneal retention of such lipid nanocarriers, modified with a complex containing boronic acid, which is able to bind with high affinity the sialic acids of mucin. The NLCs were loaded with dexamethasone and designed for the treatment of dry eye syndrome. Fluorescence marking revealed the increased retention time due to the mucoadhesive property of the nanosystem, which also proved to be a potential not irritant treatment for dry eye syndrome [111]. Another key factor that improves retention time on the ocular surface is the positive charge of nanosystems interacting with the negative charges of mucin. The addition of octa-arginine (R8) to the nanoemulsions prepared by Liu et al. imparted a positive charge to the system with the aim of increasing eye retention. Once again, C6 was used to label lipid emulsions of disulfiram. In particular, the permeation of these systems under the influence of particle size and the presence of R8 was investigated and revealed that the addition of R8 and a size of ~50 nm improved the ocular delivery performance of nanosystems. In addition, the study showed that C6 passed through the corneal epithelium mainly by paracellular pathways, but there was also a fluorescent signal in the cytoplasm, indicating a transport also by transcellular pathways [112]. The internalization of lipid nanoparticles occurs mainly through an endocytosis mechanism. This is in fact the route taken by the mRNA-based solid lipid nanoparticles prepared by Gómez-Aguado et al. The SLN were developed in order to produce IL-10 to treat corneal inflammation and was loaded with Nile Red to assess cellular uptake in corneal epithelial cells (HCE-2 cells). This platform could also be used as a theranostic model as GFP (green fluorescent protein) is produced inside the cells, so the intensity of the fluorescence is indicative of the amount of protein produced. Since GFP, once produced, remains at the intracellular level, instillation on the ocular surface of mice of the samples permitted the identification of the corneal layers where transfection occurred. All the prepared mRNA-based SLN formulations showed higher fluorescence intensity than naked mRNA, demonstrating the enhancement of their targeting ability [113]. Fluorescein is one of the most widely used fluorescent dyes for drug tracking and visualization of ocular damage following treatment. In Section 4, some clinical trials using fluorescein as a fluorescent in the study will be proposed. Fluorescein was used by Jounaki et al. for tracking vancomycin loaded NLCs. The aim of the work is the idea that NLCs for topical use could be a valid substitute of intravitreal injection in the treatment of bacterial endophthalmitis caused especially by *Staphylococcus*. Both drug-loaded and fluorescein-loaded NLCs (0.2 mg/mL) were prepared by cold homogenization technique and were used to evaluate precorneal retention with an inverted fluorescent microscope. The increased fluorescence found in the corneal epithelium demonstrated that dye-loaded, stearylamine-coated NLCs were retained more in the ocular surface. Indeed, the cationic lipid stearylamine is trapped in the mucin layer and retained due to the interaction between the fillers, facilitating the penetration and delivery of the drug to the intraocular tissues [101]. In the work of Kakkar et al., fluorescein was also used in concentrations almost like the previous work (0.25 mg/mL) to track hybrid nanoparticles. Solid lipid nanoparticles were prepared and then coated with PEG in order to encapsulate the antimycotic fluconazole. Analysis to assess the penetration into the ocular internal layers revealed that fluorescence was observed in the vitreous humor, retina, sclera and choroid after instillation of a single drop of Fluconazole-SLNs into the rat eye. In addition, the ex vivo study showed that the system exhibited a 164.64% higher flux through the porcine cornea when compared to the commercial drops ZoconVR [114]. In addition to coating the nanosystems, fluorescein was used to label them binding it covalently to the material of the nanosystem. In the work of Puglia et al. [66] an adduct is prepared between fluorescein and stearic acid named ODAF (N-(30,60-dihydroxy-3-oxospiro[isobenzofuran-1(3H),90-[9H]xanthen]-5-yl]-octadecanamide). In this case, the dye was grafted (and not loaded) and the conjugation of the lipid with the dye leads to a fluorescent probe. Solvent-diffusion technique was used to prepare SLNs of about 120 nm. The in vivo distribution from 1 h to 16 h was evaluated in rabbits and the results showed that, after ocular instillation, ODAF SLNs were mostly located in the cornea (up to 2 h), whereas over a longer time (from the second hour to the eighth hour) the fluorescent signal gradually extended toward the back of the eye, confirming the ability of controlled delivery by the lipid nanosystems [66]. Considering that the influence of blinking and tearing on ocular drug absorption was rarely evaluated in studies, Pretor et al. evaluated absorption of two lipid-based formulations, a liposome and a SLN, in presence of these two physiological conditions. The SLNs were also labeled with a fluorescent phospholipid, thus constituting another example of a grafted nanosystem. From the study, using C6 as the fluorescent compound, it is evaluated that liposomes are shown to provide a greater absorption, despite the influence of blinking (shear stress of 0.1 Pa.) and tear flow. This interesting study was carried out by coupling the use of microfluidics with channels and cultured HCE-T cells as well as the use of a fluorescent dye to simulate the physiological mechanisms; it could be useful to add this kind of assay to the basic characterization of the nanosystem addressed to ocular targets [115]. In the rhodamine family, Rhodamine B is widely available and low-cost. The following two studies promote the use of this molecule for tracking nanosystems. The first is focused on the preparation of lipid systems (niosomes vesicles) and Eudragit nanoparticles for the treatment of eye fungal infections. Encapsulation of fluconazole within these systems resulted in being a good way to increase the bioavailability of the drug compared to free drugs. The systems obtained were innovative in terms of formulation as there is a triple step: the drug was first complexed using β-cyclodextrin, then encapsulated into niosomes, and the niosomes were finally incorporated into an in-situ gelling system made by Poloxamer, HPMC and chitosan. Niosomes were labeled with Rhodamine B and then were compared to labeled polymeric nanoparticles. The fluorescent signal of CLSM analysis increased in intensity when the NPs were incorporated into the hydrogel, whereas the signal of the pure dye was limited to the superficial epithelial layers, suggesting effective permeation of the nanosystems into the inner tissues [116]. Rhodamine B was also used to study the transport of curcumin as a model drug in multilamellar liposomes. These were coated with sodium alginate grafted acrylic acid conjugated with riboflavin. These multi-dye vesicles (rhodamine and curcumin), prepared using the lipid film hydration technique, have proven to be excellent carriers for drug delivery to the retina. The study evaluated both the encapsulation efficiency of the two dyes and their in vitro release. The release test in pH 7.4 medium demonstrated time-depended release, which was faster for rhodamine than for curcumin. An extended-release profile was obtained using fluorescence, red for rhodamine and green for curcumin, showing greater entrance into the cell at 12 h than at 3 h, and greater endocytosis for smaller, more spherical particles [117].

#### 3.1.2. Polymer-Based Nanocarriers

Topical delivery of polymeric nanosystems is useful to improve corneal penetration and prolong the therapeutic response of several drugs. Nanocarriers need to be evaluated to find clinical application; specifically, their distribution in biological environment should be examined in order to understand the most appropriate strategy to address specific ocular pathologies. Plausible routes of topically instilled drug delivery for the treatment of ocular diseases involving the posterior segment include several pathways, including corneal, non-corneal and uveal routes. Successful nanocarrier development, therefore, involves fluorescent labeling useful for investigating mechanisms and biodistribution profiles of the designed systems. Polymeric nanostructures to be used as imaging diagnostic agents include various kinds of systems, such as nanoparticles, niosomes, film and nanomicelles and in-situ gel. The review of Swetledge et al. offers a detailed discussion on the biodistribution of polymer nanoparticles in major ocular tissues [118]. To improve retention time on the ocular surface, release profile and mucoadhesion performance, nanocarriers are often coated with polymers. Poly-lactide (PLA), polyglycolide (PGA), poly-lactide-co-glycolide (PLGA) and chitosan, Eudragit^®^, but also different copolymers such as PLGA-PEG, poly-(3-hydroxybutyrate-co-3-hydroxyvalerate) (PHBV) constituted by hydroxybutyrate (HB) and hydroxyvalerate (HV) and chitosan modified copolymer are some of these. Among them, many polysaccharides are used as a useful coating for nanocarriers. Some of these, including chitosan, alginate sodium, hyaluronate sodium and cellulose derivatives, are approved for ophthalmic use by the FDA and are already present in the composition of ophthalmic products on the market [119]. Depending on the type of polymer, the most suitable fluorescent probe should be chosen. A study conducted by Zhukova et al. focused on understanding the interactions between probes, polymeric nanoparticles and the biological environment. Four dyes with different degrees of hydrophobicity were encapsulated (C6, rhodamine 123, DiI) or covalently bound to the polymer (amine Cyanin 5.5, Cy5.5) in order to label PLGA nanoparticles. To increase the accuracy of the interpretation of in vivo biodistribution data, dual-labeled nanoparticles were administered, using C6 as the encapsulated label and Cy5.5 as the grafted label. Neuroimaging results showed that the signal of the nanoparticles bounded with Cy5.5 was detected in retinal vessels, whereas the signal of the encapsulated C6 was found outside of blood vessels and in tissue background. The extra vasal distribution of C6 could falsify the data interpretation, leading to erroneous assumptions that the nanoparticles could efficiently cross the blood-retinal barrier. Assessing the affinity of the dye to the polymer and the lipophilic structures could be useful in scaling up these issues. Although C6 has not proved to be an ideal label, it aided in explaining the phenomenon whereby drugs are delivered to tissues through encapsulation in nanocarriers without involving any nanoparticle penetration [120]. Similar results were obtained by Zhang et al. tracking in vivo the distribution of PLGA-NPs in the retinal blood circulation after intravenous injection. NPs were labeled with lipophilic perchlorate carbocyanins (DiI) or hydrophilic rhodamine 123 (Rho123). DiI fluorescent signal was detected for a long time (>90 min) in retinal vessels, in contrast with Rho123 whose fluorescence was short (>15 min), due to diffusion from particles and elimination from the blood circulation. To avoid artefacts, dual-labeled nanoparticles were also injected intravenously in rats. Colocalization of fluorescent markers was performed by conjugating the polymer with Cy5.5 and loading the systems with probes (DiI/Rho 123). Cy5.5 signal was detected for both cargoes in retinal vessels for more than 90 min; however, colocalization was observed only for lipophilic DiI dye, which was more closely related to the hydrophobic polymer matrix. These findings further confirm that the affinity of the dye for the polymer and cell membranes played a key role in biodistribution kinetics [121]. The hydrophilic properties of rhodamine B make it a suitable fluorescent candidate for polymers of a hydrophilic nature such as chitosan, whose mucoadhesive qualities have been exploited by X et al. for the design of topical films for the treatment of glaucoma. Corneal permeation studies demonstrated the mucoadhesive efficacy of polymeric films in transporting rhodamine B molecules through the cornea with a high permeation rate [122]. A water-insoluble derivative of the rhodamine family is rhodamine B isothiocyanate, which has affinity for hydrophobic polymers. This dye was used as a label for nanoparticles consisting of hydrophobic PHBV polymer to obtain information regarding the depth and rate of penetration after topical administration. Confocal analysis showed improved penetration deepness of encapsulated marker compared to the free one, used as a control [123]. Recently, hydrophobic C6 was doubly used as a model drug and a fluorescent marker to track surface-modified PLGA-NPs with chitosan, glycol chitosan and polysorbate 80 in retinal tissues. Tracking of NPs after topical instillation was performed by fluorescence microscopy, revealing intense staining throughout the whole eyeball, anterior segment including cornea and conjunctiva, lens, iris/ciliary body and retina, with a peak at 30 min after administration and the disappearance of the signal after 60 min. Ocular tissue autofluorescence was distinct around the outer segments of the photoreceptor. Based on the average size of the NPs (<200 nm), the specific pathway of the NPs to the retina did not exclude any of the plausible routes of delivery to the posterior segment (corneal, noncorneal or uveal pathways) [124]. C6 was also used to label polymeric nanomicelles designed for the topical treatment of fungal keratitis. The nanomicelles consisted of a chitosan oligosaccharide-vitamin E copolymer conjugated to phenylboronic acid (PBA-CS-VE) to enhance corneal retention. C6 delivery through a monolayer of HCE-T cells and 3D cell spheroids demonstrated strong corneal penetration ability. Several characteristics of the polymer were able to influence nanomicelle uptake, but the key role in the process of cellular endocytosis was attributed to the high-affinity interaction between the PBA portion and sialic acid on the surface of the cell membrane [125]. Another study using C6 as a fluorescent probe was reported by Sai et al., aiming to evaluate the corneal transportation of an in-situ gelling system based on mixed micelles. This formulation designed for curcumin was composed of micelles, consisting of 1,2-distearoyl-sn-glycero-3-phosphoethanolamine-N-[methoxy(polyethyleneglycol)-2000] (PEG-DSPE) and poly(oxyethylene) esters of 12-hydroxystearic acid (Solutol HS 15), incorporated in a gellan gum gel. Incubation of human corneal epithelial cells (HCEC) with the fluorescently labeled systems showed time-dependent and improved absorption for the encapsulated dye, compared to free C6. Transcorneal penetration was investigated in vivo by CLSM and results suggested that curcumin was able to penetrate more effectively when incorporated into the gelled systems, probably due to the increased retention time conferred by the gellan gum, which was five-fold higher than the mixed micelles alone [126]. A pilot study with C6 was performed to evaluate the feasibility of the approach in assessing the biodistribution of PLGA-PEG nanoparticles suspended in hydrogels. The preliminary study showed an important limitation due to the high green autofluorescence of the examined ocular tissues. To deal with the drawbacks highlighted by the pilot study, PLGA nanoparticles in the full study were labeled with Cy-5, a far-red fluorophore that did not overlap with the natural autofluorescence of the ocular tissues. Results from the full study showed that topical application allowed the nanoparticles to be distributed into the outer ocular tissues (cornea, episcleral tissue and sclera) and the choroid was the only internal tissue to show a slight increased fluorescence, probably attributed to the permeation of [118]. Another dye recently used as a model drug to label mucoadhesive films with a hydrophilic nature based on chitosan and poly(2-ethyl-2-oxazoline) is fluorescein sodium. To avoid precipitation of complexes between the negatively charged dye and the positively charged chitosan backbones, concentrations less than 0.1 mg/mL were used. Films tested by ex vivo (bovine cornea) and in vivo (chinchilla rabbits) studies showed excellent corneal adhesion (up to 50 min) [127]. From this review of recently published papers, it emerged that, to ascertain the applicability of nanosystems to biodistribution studies, it was necessary to (i) take in account the degree of affinity and interference between probe, polymeric carriers and cell membranes, and (ii) accurately interpret the data by selecting an effective labeling method upstream. The most reliable way to track the pathways of the systems remains the conjugation of the fluorescent dye to the polymeric core. Therefore, colocalization by double labeling may be the most appropriate technique to minimize errors in the interpretation of fluorescence signals. Currently, there is no unique approach to fluorescent polymer nanosystems that can be used for all types of labeling systems and probes.

#### 3.1.3. Metallic-Based and Inorganic-Based Nanosystems

Inorganic nanodevices became of great interest in ocular delivery due to their unique properties such as low cost, easy preparation methods, small size, tuneable porosity, high surface-volume and robust stability. Fluorescent labeling has been applied to these delivery systems to assess their ability to cross ocular barriers and provide therapeutic efficacy [128]. Corneal barrier functions were investigated by Mun et al. using two types of silica nanoparticles (thiolate and PEGylated) fluorescently labeled with 5-(iodoacetamido)-fluorescein (5-IAF). Permeation studies were performed in vitro on intact or β-cyclodextrin pretreated bovine corneas. To provide experimental parameters close to in vivo conditions and to avoid artifacts such as the potential risk of corneal swelling when using Franz diffusion cells, the “whole-eye” method was used. 5-IAF-loaded thiolate silica nanoparticles, PEG-grafted silica nanoparticles (5-IAF-PEG), sodium fluorescein and fluorescein isothiocyanate dextran solutions were tested. It resulted that fluorescein salt (376 Da) did not uniformly penetrate the cornea; however, the dye was detected in the stroma. Larger molecules such as FITC-dextran (400 Da) and 5-IAF-PEG formed a layer on the corneal surface with no permeation of the epithelial membrane. Β-cyclodextrin pre-treatment disrupted the integrity of the cornea by providing homogeneous permeation of the low-molecular-weight dye, although it did not improve the penetration of larger molecules. Concerning NPs, no permeation was reported regardless of surface modification, particle size and pre-treatment with β-cyclodextrin, thus suggesting that the tight junctions of the corneal epithelium acted as the main barrier to permeation. The absence of penetration and confinement on the corneal surface was observed for thiolated NPs because of the formation of disulfide bonds between the NPs thiol groups and the cysteine domains of the mucus glycoprotein layer. The interaction between mucin and -SH thiol groups remained a limiting permeation factor even after the removal of the epithelial layer. NPs PEGylation was able to mask thiol groups, allowing passage into the stroma [129]. Baran-Rachwalska et al. designed a novel platform consisting of hybrid silicon-lipid nanoparticles, aiming to deliver siRNA to the cornea by topical administration. A fluorescent oligonucleotide duplex, siRNA transfection indicator (siGLO), was employed as a tracking probe to assess in vitro cellular uptake on a human corneal epithelial cell line (HCE-S) and in vivo corneal penetration on wild-type mice. Red fluorescence of the oligonucleotide marker allowed detection of nanoparticles in all layers of the cornea 3 h after instillation, in contrast to the control siGLO. The tracking of biodegradable nanosystems in corneal tissues was confirmed by the reduction of protein expression in the corneal epithelium, making them ideal candidates for therapeutic oligonucleotide delivery [130]. Biodegradable mesoporous silica nanoparticles (MSNs) loaded with carboplatin were designed by Qu et al. for the treatment of retinoblastoma. Carboplatin, being an anticancer drug, causes severe side effects; therefore, it is necessary to focus the action strictly on the target site. For this purpose, MSNs were surface modified by conjugation with an ideal target, epithelial cell adhesion molecule (EpCAM), in order to increase specificity as well as therapeutic efficacy. To assess the targeting efficacy of the designed systems, the authors evaluated the cellular uptake of untargeted and targeted MSNs in retinoblastoma Y79 tumor cells. Rhodamine B and Lysotracker Green were used as fluorescent probes to track cellular and subcellular uptake of the vectors. Increased cellular uptake for targeted MSNs was attributed to EpCAM-specific receptor-mediated cellular internalization. Lysosomal localization of MSNs confirmed that the nanosystems followed the endocytosis pathway for drug delivery [131]. A hexa-histidine with metal ions nanosystem was designed to deliver Avastin in the treatment of corneal neovascularization (CNV). Pre-corneal retention time and ability to cross ocular barriers were studied on a rat CNV model induced by alkaline burns by FITC labeling the systems. Avastin encapsulated in the vectors showed a longer precorneal adhesion time compared to the free drug. These innovative systems have emerged as a promising platform for ocular topical delivery of protein drugs [132]. An interesting zirconium-porphyrin metal-organic framework (NPMOF) has been designed for drug tracking and delivery. The bright fluorescence self-emitted by the metal-organic framework qualifies the carriers to be applied for imaging. NPMOF was used as a skeleton for the delivery of methylprednisolone, a very efficacious corticosteroid in the treatment of retinal degenerative diseases. Adult zebrafish with photoreceptor degeneration induced by high-intensity light exposure were used to test in vivo distribution and therapeutic efficacy. Red fluorescence signals were detected in choroid, retina, photoreceptors and retinal pigment epithelium for up to 7 days. Recovery of visual function by rapid regeneration of photoreceptors and proliferation of Müller’s glia and retinal regeneration were reached after a single intravitreal injection. NPMOF vectors represent a novel delivery system for the treatment of diseases affecting the posterior eye segment [133].

#### 3.1.4. Protein-Based Nanosystems

Protein-based nanosystems have attracted considerable interest in recent years and are designed for drug delivery, diagnostics and bioimaging. These highly bio-compatible systems, which have been extensively studied in the biomedical field, owe their properties to the protein they are composed of. Among the proteins used in their preparation, there are antibodies, enzymes, animal and plant proteins, collagen, plasma proteins, gelatin and proteins derived from virus capsids [134]. Fluorescent proteins are usually used to monitor protein-protein interactions, protein localization and gene expression. However, without any carrier, the fluorescent efficiency of a single protein is relatively low. The use of fluorescent protein-labeled nanomaterials improves loading due to increased surface area and allows the development of fluorescent nanosystems useful in bioimaging and biosensing. In the study carried out by Yang et al., nanoparticles were prepared from regenerated silk fibroin. This protein, which is the most abundant in silk, is considered to have high biocompatibility and degradability properties. In the biomedical field, it has been used for drug delivery in small nanosystems, biological drug delivery, gene therapy, wound healing and bone regeneration. The formulation is targeted for intravitreal injection with the aim of increasing the bioavailability of the drug in the retina. Fluorescein isothiocyanate labeled bovine serum albumin (FITC-BSA) has been encapsulated as a model drug. In vitro cytotoxicity studies were conducted on ARPE-19 cells, showing that these nanosystems were very compatible. In addition, in vivo comparison of the biodistribution in posterior ocular tissues in rabbits revealed increased retention in the retina due to encapsulation in the nanosystem rather than with a solution of model drug [135,136]. Albumin is widely used in the preparation of ocular nanosystems [137]. In a recent study, bovine serum albumin nanoparticles loaded with apatinib were prepared for the treatment of diabetic retinopathy. In contrast to the previous study, in this disease, invasive administration has to be avoided, so topical administration is the ultimate goal. The nanoparticles were coated with hyaluronic acid (HA) to increase mucoadhesion. The biodistribution study in retinal tissue was carried out by preparing fluorescent nanosystems with 1,1′-dioctadecyl-3,3,3′,3′-tetramethylindodicarbocyanine, 4-chlorobenzenesulfonate salt (DiD) solution in ethanol (0.5 mg/mL), which was added during the formulation phase. Through the comparative in vivo biodistribution study, it was shown that HA-coated nanoparticles demonstrate higher fluorescence in retinal tissue compared to uncoated nanoparticles, thus representing a viable alternative to intravitreal injection, maintaining comparable perfusion and bioavailability [138]. Another study involved the preparation of nanoparticles using pseudo-proteins for the potential treatment of ophthalmic diseases. Ten types of nanoparticles obtained by precipitation of pseudo-proteins were prepared, then they were loaded, and some of them were also pegylated; finally, they were labeled with a fluorescent probe, fluorescein diacetate (FDA) or rhodamine 6G (Rh6G), to assess ocular penetration. Corneal fluorescence was obtained as expected, while surprising results were the reaching of tissues such as the sclera and retina. Thus, they proved to be a promising delivery system for topical use in chronic eye diseases [139].

### 3.2. Diagnostics

Labeling nanoparticles with fluorescent probes was demonstrated to be a useful approach to improve the effectiveness of some diagnostic tests aimed to detect ocular pathologies. In fact, some eye diseases require a prompt diagnosis in order to contain possible damages related to the ongoing pathways involved. Age-related macular degeneration (AMD) is the main cause of vision loss for over-65-year-olds [37]; this pathology has often been analyzed to improve diagnostic techniques since it has several predisposing factors, and early detection is crucial to avoid degeneration toward blindness [140]. AMD has an unclear etiology, although oxidative stress is considered one of the main risk factors [141]; as a matter of fact, clinical studies demonstrated the importance of supplementation with antioxidants in order to slow down the progression of AMD [142,143]. Physiological antioxidant patterns involve metallothioneins (MT), low molecular mass proteins characterized by the presence of cysteine sulfur ligands, which are able to scavenge free radicals, thus protecting cells and tissues. The retina is particularly subject to oxidative stress due to visible and UV light exposure; moreover, age progression involves a reduction of MT expression, predisposing to AMD [144]. For this reason, bioimaging these proteins in ocular tissues could be an important tool useful to highlight the tendency to develop AMD. For this purpose, fluorescent gold nanoclusters involving Cu and Zn and bioconjugated with specific primary antibodies were developed by Cruz-Alonso and coworkers [145]. Laser ablation (LA)-inductively coupled plasma (ICP)-mass spectrometry (MS) technique was used to identify ^63^Cu^+^ and ^64^Zn^+^ in the retina of post-mortem donors since MT bind both Cu and Zn [146]. This method showed results comparable with conventional immunohistochemistry for MT proteins, with amplification of signals related to the presence of nanoclusters, which allowed the obtainment of higher resolution bioimages. An in vivo model of human “wet” AMD is laser-induced choroidal neovascularization (mouse LCNV) mouse, in which the inflammatory biomarker vascular cell adhesion molecule-1 (VCAM-1) is highly expressed. Gold nanoparticles functionalized with anti-sense DNA complementary to VCAM-1 mRNA were developed by Uddin et al., who aimed to detect this molecule, thus assessing the occurrence of oxidative stress [147]. The fluorescence in-situ hybridization (FISH) technique was used to perform photothermal-optical coherence tomography (PT-OCT) involving a fluorescent probe (Alexafluor-647) bonded to 3′ end of anti-sense DNA in order to highlight its interaction with the target mRNA. The conjugation of anti-sense DNA to gold nanoparticles proved to protect from the degradation performed by DNase while enhancing the uptake, probably through endocytosis, as suggested by transmission electron microscopic (TEM) images of retinal cells; moreover, it was verified that no inference in the fluorescence was produced due to low pH, which is characteristic of inflamed tissues. Compared to the control group, in vivo systemic injection in mice confirmed the enhancement in the fluorescent signal for anti-sense DNA coupled with nanoparticles, which mostly depended on VCAM-1 mRNA hybridization, thus demonstrating the potentiality of the developed platform as a tool to obtain direct images of endogenous mRNA in a tissue. In some cases, this pathology requires transplantation of photoreceptor precursors (PRPs) in the subretinal space, which was successfully performed, guaranteeing a certain vision restoration [148]. For a certain period, monitoring of the efficiency of the transplantations needs to be performed. As confirmed by Chemla and coworkers [149], gold nanoparticles could be transplanted together with photoreceptor precursors cells labeled with a fluorescent probe (Alexa 594) in order to ameliorate the efficiency of computed tomography (CT) and optical coherence tomography (OCT) in assessing the success of the transplant. The nanoparticles were firstly characterized in order to assess their safety, thus demonstrating no toxicity toward the transplanted cells and no occurrence of inflammation in the retina and vitreous. Furthermore, this platform demonstrated to enhance X-ray signal detected by CT and related to cell survival without interference from the particles secreted from the cells [150]; moreover, they were also able to increase optical signal for OCT by up to 1.4-fold and track cells migration toward layers deeper than the injection site. These results confirm the efficiency of such a platform in the monitoring of transplantation but also suggest a potential use for ameliorating existent molecular imaging in cell therapy and diagnostic. Another important diagnostic test is fundus fluorescein angiography (FFA), which allows highlighting vascular leakages in retinal and choroidal pathologies [151]. This clinical tool is useful to diagnose several ocular diseases: age-related macular degeneration, which is characterized by hemorrhaging and exudation in the retina [140]; diabetic retinopathy, which involves retinal damages related to microvascular modification which are clinically not revealable in the early stages [152]; diabetic macular edema, whose pathophysiology implicates modifications of choroidal and retinal vasculature due to BRB impairment [153]. Furthermore, the aforementioned diseases are characterized by alterations of ocular vessels, and share the consequent compromission of visual activity, if not quickly detected and treated. Fluorescein sodium (FS) is injected intravenously to perform this analysis, diffusing in the blood vessels, thus allowing us to observe them through a confocal scanning laser ophthalmoscopy system. Despite it being considered relatively safe, nausea and vomiting frequently occur, while severe effects such as anaphylaxis are rare. The main drawbacks are the diffusion of FS into normal tissues and cellular absorption, with long retention, which were overcome using nanoparticles. Cai et al. coworkers developed a high molecular weight polyethyleneimine (PEI) nanoparticles which demonstrated to successfully couple fluorescein [151]; moreover, in vitro studies showed good cytocompatibility, no significant difference in apoptosis rates considering various concentration tested, no genotoxicity, and no morphological changes or significant difference in endothelial tube formation. Cellular uptake assays, carried on with different concentrations of free FS and FS-NP, confirmed similar rapid uptake by cells, with a concentration-dependent and time-dependent fluorescence of main retinal vessels and microvessels. Furthermore, free FS was longer retained in cells when compared to FS-NP, as highlighted by in vivo fluorescence studies, suggesting a potential decrease in FS toxicity. These results confirm the potentiality of this platform as a diagnostic tool to detect retinal vessels; moreover, PEI enhances fluorescein metabolism, thus reducing its toxicity. Other polymeric nanoparticles developed as a potential diagnostic tool are composed of copolymerized glycerol mono methacrylate (GMMA), glycidyl methacrylate (GME) and ethylene glycol dimethacrylate (EGDMA), which were functionalized with Vancomycin, Polymyxin B or Amphotericin B, in order to detect the presence of Gram-positive bacteria, Gram-negative bacteria and fungi through a specific bond with the respective antibiotic or antimycotic [154]. The occurrence of such bonds was differently highlighted using fluorescent Vancomycin, and probes such as fluorescein isothiocyanate (FITC) and Calcofluor White. Tests conducted on various microbiological strains showed a proportional increase in the fluorescence signal with the increase in the number of organisms involved; moreover, the presence of functionalized polymers favored the microorganism bonding. Besides the biocompatibility of this platform, another advantage of this platform is the possibility to be shaped as a contact lens requiring only a 30-min exposure to efficiently detect the occurrence of infection, thus demonstrating to be a promising approach for an easy diagnosis of corneal infections.

### 3.3. Nanotheranostics

**Figure 5:**
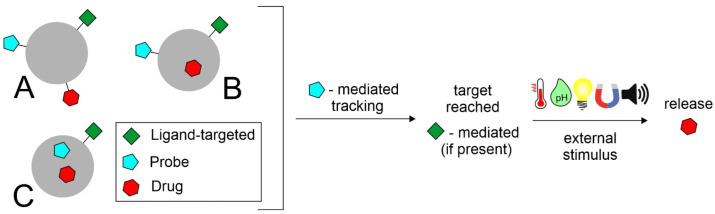
Prototypes of theranostic nanosystems and their mechanism of action. In figure: (**A**) labeling of both probe and drug; (**B**) loading of drug and labeling of probe; (**C**) co-loading of drug and probe.

The recent development of systems that integrate the treatment of diseases with their diagnostics is referred to as theranostics. When the system is in a nanoscale range, it is called nanotheranostics. Figure 5 shows prototypes of nanosystems suitable for theranostic purposes.

The development of these applications has given researchers a new way of diagnosing and treating diseases such as cancer, diabetic retinopathy and age-related macular degeneration [37,155]. Among the major chronic eye diseases, diabetic retinopathy is the most prevalent. Angiogenesis in the posterior eye segment is the main cause of retinal impairment. Clinical management consists of pathological diagnosis and intravitreal injections of vascular endothelial growth factor (VEGF) inhibitors to suppress neovascularization. The development of innovative nanotheranostic systems is emerging to overcome these critical problems with less invasive methods to diagnose and treat ocular angiogenesis synergistically. Silicon nanoparticles conjugated to the peptide Cyclo-(Arg-Gly-Asp-d-Tyr-Cys) (c-(RGDyC)) (SiNP-RGD) were designed by Tang et al. with the dual action of imaging and treating ocular neovascularization. The effective anti-angiogenic capability of these biocompatible theranostic nanoprobes was based on the combination of a specific detection by labeling endothelial cells and angiogenic blood vessels and a selective inhibition of neovascularization [156]. Metal NPs are receiving a lot of attention as carriers for the delivery of biomolecules, among which silver NPs (AgNPs) have found numerous applications. Stati et al. designed curcumin stabilized AgNPs using a green and cost-effective method to exploit the promising characteristics of this polyphenol in the in vivo treatment of human pterygo. Curcumin is a molecule suitable for theranostic application, as widely reported in the work of Shabbir et al. [157]. Pterygo is a progressive eye disease that could culminate in an irreversible impairment of visual function. Available treatments require invasive surgical procedures, such as excision, which often leads to a worsening of the clinical picture. Spectroscopic techniques revealed a strong plasmonic resonance between the silver nuclei and the curcumin molecule, demonstrating the presence of the polyphenol on the surface of AgNPs. The biological efficacy of the formulation was tested in vitro on human keratinocytes derived from pterygium explants, showing decreased cell viability in treated samples compared to controls. Although no studies have been conducted to track the fate of NPs, the fluorescent emission of the samples could be exploited for bioimaging applications [158]. Fluorescent silicon nanoparticles modified with Vancomycin were designed by Zhang et al. for the simultaneous non-invasive diagnosis and treatment of keratitis induced by Gram-positive bacteria. These nanotheranostic agents have demonstrated, in combination with strong antimicrobial activity against *Staphylococcus aureus*, a rapid (<10 min) imaging capability both in vitro and in vivo. The rapidity with which bacterial keratitis was diagnosed at an early stage suggests that these devices may be useful in preventing the progress of the disease, which could impair visual function if not treated [159]. Oliveira et al. designed hybrid theranostic systems consisting of a lipid matrix of 1,2-dipalmitoyl-sn-glycero-3-phosphatidylcholine (DPPC), coated with Pluronic^®^ F127, covalently bound with the fluorescent probe 5(6)-carboxyfluorescein and loaded with the photosensitizing agent verteporfin. Preliminary studies on a glioblastoma cell line (T98G) were conducted to evaluate the potential application as theranostic nanodevices. The fluorescence of the systems revealed on the cancer cell membrane and the 98% reduction in cell viability of T98G cells encouraged further investigation of such multifunctional platforms for the treatment and diagnosis of ophthalmic diseases [160]. Photothermal therapy has been making inroads into the eye sector for a couple of years now. Heat therapy refers to the use of heat as a therapeutic tool to treat diseases such as tumors. In the recent work of Li et al., an approach to treat choroidal melanoma using nanocomposites was designed. Nanosystems were synthesized based on hydrogel, which is itself based on rare-earth nanoparticles. These platforms emit fluorescence in an NIR-II region. Characterized by their tiny size of less than 5 nm, they are targeted for the treatment and simultaneous bioimaging of choroidal melanoma. They have been incorporated into biodegradable hydrogels based on PNIPAM dual response, which could release the drug in a controlled manner by responding to heat and glutathione in the tumor microenvironment. The nanocomposites were then further decorated with indocyanine green (ICS) and folic acid (FA) to enhance therapeutically and to target specificity and the possibility of achieving photothermal therapy [161]. A lot of studies showed the potential of therapeutic contact lenses in the management of eye disease [162]. Infectious endophthalmitis is a growing concern that causes irreversible damage to intraocular tissue and the optic nerve. The work of Huang et al. focuses on the design of contact lenses consisting of hybrid hydrogels based on quaternized chitosan composite (HTCC), silver nanoparticles and graphene oxide (GO). Fungal keratitis infection often leads to the formation of a biofilm, which is particularly difficult to be penetrated by antifungal agents, especially through eye drops. In addition, the bioavailability of a drug such as Voriconazole is very limited. The function of these nanoparticles is not only to deliver Voriconazole in the treatment of fungal keratitis, but also to act as an antimicrobial agent due to its properties. In fact, the materials used, such as quaternized chitosan, have inherent antimicrobial capabilities. The dual functionality makes this system a useful theranostic approach for the treatment of eye infections [163]. The study by Jin et al. reports a therapeutic nanoplatform based on UiO-66-NH_2_ to combine photodynamic therapy (PDT) and targeting lipopolysaccharides (LPS) through polypeptide modification (YVLWKRKFCFI-NH_2_). The fluorescent used was Toluidine blue (TB), which acted as a photosensitiser (PS) and was loaded into UiO-66-NH_2_ nanoparticles (NPs). The dye acts both as a tracer and as a therapeutic agent through photodynamics. The release of the fluorescent is pH-dependent. The study proved beneficial against *Pseudomonas aeruginosa* and *Staphylococcus epidermidis*, and the in vivo model showed positive results in the treatment of endophthalmitis [164].

## 4. Fluorescent Status for Ocular Therapies in Clinical Trials and Market

Scientific progress in the field of ocular nanomedicine is constantly advancing, many nanoformulations for the treatment of ophthalmic diseases have been clinically investigated, and some have already been introduced to the market. A list of nanomedicines for eye diseases in clinical trials and approved by the Food and Drug Administration (FDA) is discussed in the review provided by Khiev et al. [165].

Novel nanosystems on the market included NorFLO, a dietary supplement based on a patented curcuma-phospholipid formula (iphytoone^®^). Phospholipids enhanced the targeted distribution of curcumin in the eye, and the efficacy of the formulation has been demonstrated in over 40 studies in processes triggered or sustained by chronic inflammation, found to be the cause of many eye diseases. Prolidofta is another supplement marketed as an ocular spray to counteract inflammatory processes affecting the palpebral component and restore any functional and structural changes. This spray consists of small vesicles (50–500 nm) made up of a double layer of phospholipids surrounding an aqueous core for the delivery of vitamins A and E. OMK1-LF is an ophthalmic liposomal solution based on citicolin, an endogenous molecule that restores the damage caused by glaucoma in the cell membranes and hyaluronic acid, which acts to hydrate, protect and lubricate the tear film. TriMix is an eye drop with cross-linked Hyaluronic Acid, Trehalose and Stearylamine Liposome indicated to counteract dryness and eye irritation.

Regarding imaging in surgery, near-infrared fluorescence (NIRF) with the dye indocyanine green has been widely used. Indocyanine green (ICG) is a clinically approved NIRF dye in ophthalmology for imaging retinal blood vessels; an overview of surgical applications using indocyanine green fluorescence imaging has been proposed by Alander et al. [166]. Based on clinicaltrials.gov, a website database of clinical trials conducted around the world (as accessed on 1 April 2022), since 2010 fluorescence imaging has been used in clinical trials to assess the integrity or damage of ocular surfaces after administration of novel nanosystems. Green dye fluorescein was used in 13 clinical trials for the evaluation of nanosystems with different ocular indications, from dry eye to autoimmune Sjögren’s syndrome. The role of the dye and details of the studies are given in Table 3.

Fluorescence for the development and clinical investigation of innovative ocular nanosystems seems to be a promising strategy to increase the number of formulations able to reach market commercialization. In Table 4, few products with fluorescein approved by the FDA are reported.

## 5. Challenges and Future Perspectives

The growing number of people who have blindness and visual impairment indicates a continuous increase in the need for care and treatment. Given this evidence, urgent action is required to address this largely preventable global problem and provide adequate eye care services. There are still many gaps in the literature regarding optimal design and traffic pathways within the eye. In particular, further research is needed to unravel the transport mechanisms across certain barriers in the eye. Moreover, rapid clearance remains a challenge for nanosystems as they need to release their payload before being eliminated from the eye. Many studies focus on assessing the distribution in various tissues once the formulation has been instilled into the eye [106,107,108,109,110,111,112,113,114,115,116,117,118,119,120,121,122,123,124,125,126,127,128,129,130,131,132,134,137,138,139]. Unfortunately, few studies focus on assessing how mechanisms including blinking, tear drainage and ocular metabolism may interact with nanosystems [66,115]. Among other things, a very important aspect is the evaluation of the toxicity and the actual applicability of these systems. In fact, many of them are quite complex, and the applicability, especially in the theranostic field, is not entirely easy. The evaluation has to be as precise as possible because many eye studies use rodent models; this is highly questionable, especially in the quantification of distribution and kinetic properties of nanoparticles in the eye, as there are many significant differences between the rodent and human eye. Therefore, the most impactful future studies on this topic will come from larger animal models with eyes that are physiologically and anatomically more similar to ours.

The increasing use of fluorescent probes in the realization of biosensors for colorimetric and radiometric identification of specific targets is a great step forward since the fluorescence represents a non-invasive diagnostic method. This has important benefits in early diagnosis through self-medication screening based on membranes or other platforms containing the appropriate fluorescent probe. These tools are also applicable in epidemics through the realization of specific self-tests based on ELISA or other strategies able to identify the etiological agent selectively. A large and growing field is the use of these probes as part of theranostic photo switch structures, able to change their structure after light stimulus, releasing the therapeutic agent and activating or switching off the fluorescence of the probe. Thus, fluorescence allows accurate and quantitative identification (under certain conditions even by the naked eye as also through in vitro tests) of the drug release process. Therefore, the use of fluorescent probes is finding increasing use in experimental and advanced ocular chemotherapy using photo-activated systems.

## 6. Conclusions

The eye has a complex anatomical structure, representing the main difficulty for drugs to achieve this target. Nanomedicine has made it possible to overcome several difficulties related to the administration of this almost isolated compartment. The study of the pathways followed by the nanosystems makes it possible to assess the effective achievement of the target site and to consider any non-productive distribution in undesirable tissues with the possible onset of side effects. The biodistribution study also allows the correlation between the chemico-physical parameters of the nanosystems (e.g., ZP, size, morphology, mucoadhesive properties, etc.) and the paths followed by them. This investigation is also aimed at evaluating and developing strategies to bypass physiological barriers of the eye, including tight junctions, tearing and blinking, that could compromise targeting effectiveness. The development of bioimaging mediated by fluorescent probes has improved the efficiency of some diagnostic tests for eye diseases. It is known that early (or rather preventive) diagnosis is a necessity to limit the damage, especially in the long term, caused by specific diseases. The involvement of fluorescent nanoparticles as diagnostics demonstrated to be suitable for detecting the occurrence of pathological pathways, ameliorating techniques already employed in ocular diagnostic, thus providing better results through equipment of common use (OCT, CT, FFA, etc.). This is where the important contribution of fluorescent probes to nanotheranostic approaches becomes relevant since, in these systems, diagnostic and therapy coexist. Tracking the nanoparticles makes it possible to highlight the effective achievement of the target, thus following the release of the therapeutic agent through an external stimulus (e.g., ultrasounds, magnetic fields, light, etc.). In conclusion, as highlighted in this review, the potential applications of fluorescence in the ocular field have been demonstrated as a useful strategy for translating nanoformulations into marketable drug candidates. In addition, to the best of our knowledge, there are no reviews focused on this topic, so this work aims to raise awareness and summarize the use of fluorescents in the ocular field.

## Figures and Tables

**Table 1:** Conventional route of ocular delivery: benefits and limits.

Administration Route	Benefits	Limits	Ocular Anterior/Posterior Target	References
Oral	Non-invasive. Increased compliance.	Difficult achievement of the anterior and posterior tracts of the eye. Possible degradation by digestive fluids. Possible low absorption and bioavailability. Hepatic first-pass metabolism. Presence of anatomical barriers (blood-aqueous barrier and the blood-retinal barrier).	Potentially both	[45,46,47,48]
Systemic (Intravenous and intramuscular)	Avoided first-pass metabolism.	Difficult achievement of the anterior or posterior segment of the eye. Lower compliance. Presence of anatomical barriers (blood-aqueous barrier and the blood-retinal barrier). Sterility of the final form	Potentially both	[48,49]
Parenteral (intravitreal, subretinal, suprachoroidal, subconjunctival, intracameral, intrascleral, and intrastromal)	Deposit of the therapeutic agent in the eye, in some cases directly at the site of action. Increased local concentration of the drug. Reduced required dose and avoided off-target actions. Bypassing of ocular epithelium and other barriers, resulting in increased bioavailability.	Administration performed by specialized personnel. Invasive technique. Short-term complications, including retinal damage, endophthalmitis, haemorrhage, intraocular inflammation, and increased Intraocular Pressure (IOP). Sterility of the final form	Posterior	[50,51,52,53,54,55]
Topical	Over 90% of the ophthalmic product on the market.	Rapid precorneal elimination of the drug due to eyelid reflex, tear drainage, dilution by tears, and systemic absorption from the conjunctival sac. Misapplication of the product to the ocular surface. Presence of corneal epithelial barrier. Narrow barriers at the front and back of the eye (limit and regulate fluid and solute uptake). Complex kinetic processes of absorption, distribution and elimination, influenced by physiology, the physicochemical properties of the drug (lipophilicity, charge, size and shape of the molecule) and the formulation (pH, buffer, tonicity, viscosity, possible presence of preservatives and stabilizers). Allowed permeation of small lipophilic molecules through the cornea and of larger or hydrophilic compounds through the conjunctiva and the sclera. Achievement of the anterior segment for only 1% of the administered dose segment, and an even smaller percentage to the posterior segment. Sterility of the final form	Both	[56,57,58,59,60,61,62,63,64]

**Table 2:** Physico-chemical properties of the main fluorescent probes used in ocular bioimaging.

Probe	Chemical Structure	Molar Mass (g mol^−1^)	Solubility in Water	Excitation (nm)	Fluorescence (nm)
Coumarin-6	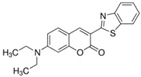	350.43	Insoluble	488–666	502–649
Curcumin	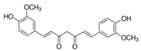	368.38	Insoluble	300–470	571
Cyanine 5-phosphoramidite	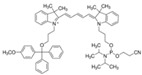	944.21	Insoluble	649	666
1,1′-dioctadecyl-3,3,3′,3′-tetramethylindocarbocyanine perchlorate	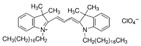	933.87	Low	550	565–588
Fluorescein	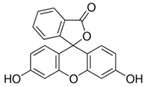	332.31	Insoluble	465–490	494
Fluorescein sodium salt	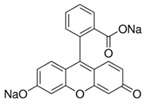	376.27	Soluble	460	512
5-aminofluorescein	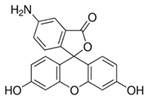	347.32	Soluble	450–490	500–550
Fluorescein-5-isothiocyanate	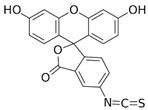	389.38	Insoluble	495	519
5-(iodoacetamido)fluorescein	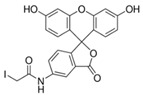	515.25	Insoluble	492	518
5(6)-carboxyfluorescein	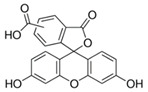	376.32	Low	495	520
Nile Red	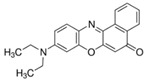	318.37	Insoluble	543–633	550–700
Rhodamine B	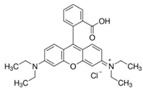	479.01	Soluble	488–530	600–633
Rhodamine B isothiocyanate	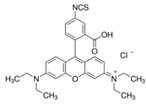	536.08	Insoluble	553	563–650
Rhodamine 123	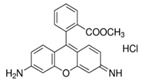	380.82	Low	488	515–575
Rhodamine 6G	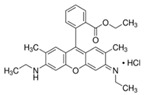	479.01	Soluble	480	530
Toluidine Blue O	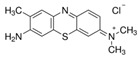	305.83	Soluble	595	626

**Table 3:** Use of Fluorescein dye in clinical trials of drug delivery systems for eye diseases.

Role of Molecule in the Study	Name and Type of Formulation Tested	Name of the Study	Pathologies	Status	Identified Number of the Study
Evaluate corneal and conjunctival damage	LAMELLEYE Liposomal suspension	Lamelleye vs. Comparator for the Treatment of Dry Eye Disease	Dry Eye Syndromes	Completed	NCT03052140
Evaluate tear break up time and corneal damage	AQUORAL LIPO (liposomal solution) in contact lens	Efficacy of “Aquoral Lipo” Artificial Tears in Contact Lens Wearers With Discomfort	Contact Lens Complication	New study (March, 2022) not yet recruiting	NCT05290727
Evaluate corneal and conjunctival damage	LAMELLEYE Liposomal suspension	LAMELLEYE for the Treatment of Dry Eye Symptoms in pSS Patients	Primary Sjögren Syndrome	Unknown	NCT03140111
Evaluate corneal damage	LIPOSIC AND TEARS NATURALE FORTE (liposomal suspension)	Comparison of the Effects of Two Tear Substitutes in Patients with Dry Eye Syndrome	Dry eye	Completed	NCT03211351
Evaluate ocular surface damage	TEARS AGAIN (liposomal spray)	Dry Eye Treatment with Artificial Tears	Dry eye	Completed	NCT02420834
Evaluate the absence of anterior chamber cells	OCS-01 (Dexamethasone Cyclodextrin Nanoparticle Ophthalmic Suspension 1.5%)	OCS-01 in Treating Inflammation and Pain in Post-cataract Patients (SKYGGN)	Inflammation and pain following cataract surgery	Completed	NCT04130802
Evaluate corneal damage	Intravenous Administration of Secukinumab (AIN457) or Canakinumab (ACZ885) solution	The Effects of a Single Intravenous Administration of Secukinumab (AIN457) or Canakinumab (ACZ885) in Dry Eye Patients	Dry eye	Completed	NCT01250171
Evaluate corneal and conjunctival damages	Tanfanercept (HL036) Topical Ophthalmic Solution	A Study to Assess the Efficacy and Safety of Tanfanercept (HL036) Ophthalmic Solution in Participants With Dry Eye (VELOS-3)	Dry eye	Recruiting. Phase III	NCT05109702
Evaluate conjunctival damage	HL036 0.10 percent (%) ophthalmic solution as topical ophthalmic drops	A Study to Assess Efficacy of HL036 in Subjects With Dry Eyes (VELOS-1)	Dry eye	Completed. Phase II	NCT03334539
Evaluate changes in inferior cornea	NCX 4251 (fluticasone propionate nanocrystal)	Study Evaluating the Safety and Efficacy of NCX 4251 Ophthalmic Suspension for the Treatment of Blepharitis	Blepharitis	Completed	NCT04675242
Evaluate tear film break-up time	SYSTANE^®^ Complete Nanoemulsion ocular lubricant (Propylene glycol-based eye drops)	Study of Efficacy and Tolerability of SYSTANE Complete in Patients with Dry Eye Disease	Dry eye	Completed	NCT03492541
Evaluate corneal damage	TJO-087 Cyclosporine ophthalmic Nanoemulsion (0.08%)	Evaluating the Efficacy and Safety of TJO-087 in Moderate to Severe Dry Eye Disease Patients	Dry eye	Recruiting	NCT05245604
Evaluate corneal damage	OCU300 Brimonidine Tartrate Nanoemulsion	Study of Brimonidine Tartrate Nanoemulsion Eye Drops in Patients With Ocular Graft-vs-Host Disease	Ocular Graft Versus Host Disease	Completed	NCT03591874

**Table 4:** FDA-approved products with fluorescein.

Name	Active Ingredients	Company	Description	NDA
Altafluor Benox	Benoxinate Hydrochloride; Fluorescein Sodium (0.4%; 0.25%)	Altaire Pharms Inc. (Aquibogue, NY, USA)	Solution/Drops; Ophthalmic	208582
Fluorescein Sodium And Benoxinate Hydrochloride	Benoxinate Hydrochloride; Fluorescein Sodium (0.4%; 0.3%)	Bausch & Lomb (Dublin, Ireland)	Solution/Drops; Ophthalmic	211039

## Data Availability

Not applicable.
